# Effect of Hypertension on EPR effect Induced by Polymer Nanomicelles in Renal Cell Carcinoma in vitro

**DOI:** 10.12669/pjms.39.1.6584

**Published:** 2023

**Authors:** Zhenyu Cui, Tao Ma, Wenzeng Yang, Weibing Shuang

**Affiliations:** 1 Zhenyu Cui, Shanxi Medical University, Taiyuan, 030001 Shanxi, China, Department of Urology, Affiliated Hospital of Hebei University, Baoding, Hebei, 071030, China; 2 Tao Ma, Department of Urology, Affiliated Hospital of Hebei University, Baoding, Hebei, 071030, China; 3 Wenzeng Yang, Department of Urology, Affiliated Hospital of Hebei University, Baoding, Hebei, 071030, China; 4 Weibing Shuang, Department of Urology, The First Hospital of Shanxi Medical University, Taiyuan, 030001 Shanxi, China

**Keywords:** Hypertension, Polymer nanomicelles, Renal cell carcinoma, EPR effect

## Abstract

**Objective:**

To investigate the effect of hypertension on the (enhanced permeability and retention, EPR) effect induced by polymer nanomicelles in renal cell carcinoma in vitro.

**Methods:**

A total of 80 patients with renal cell carcinoma treated at the Department of Urology Surgery in the Dept. of Urology of the Affiliated Hospital of Hebei University from Oct. 2019 to Oct. 2020, were analyzed retrospectively. The hypertension group (experimental group) included 40 patients, and the normal blood pressure group (control group) included 40 patients. The diagnosis of renal clear cell carcinoma was confirmed by preoperative auxiliary examinations, such as ultrasonography and CT combined with postoperative pathological analysis. All patients underwent laparoscopic radical nephrectomy for renal cell carcinoma. Polymer nanomicelles (loaded with prolonium iodide) were perfused into the resected kidney specimens within the specified time. The iodine enrichment of polymer nanomicelles in renal tumors was assessed by CT scanning. The peak EPR effect and the time to the peak were statistically compared between the two groups.

**Results:**

No significant differences were found in age, sex, location of kidney disease, tumor location or tumor size between the two groups (*p>* 0.05). The peak (*χ̄*±*S*) of the EPR effect in experimental group was 3.60±0.95 ug/cm^3^ and 3.01±0.96 ug/cm^3^ in control group, respectively. There was significant difference between the two groups (*p<* 0.05). The time to the peak of the EPR effect was 3.76±0.75 h in experimental group and 3.82±0.93 hour in control group, respectively. No statistically significant difference was found in the time to the peak of the EPR effect between the two groups (*p>* 0.05).

**Conclusion:**

Hypertension has a certain effect on the EPR effect induced by polymer nanomicelles in renal cell carcinoma in vitro.

## INTRODUCTION

Renal cell carcinoma (RCC) is the most common malignant tumor of the renal parenchyma. Over the past 20 years, RCC has been ranked the second most common urinary system tumor, with an average annual increase in incidence of 6.5%.^1^ There are many types of RCC, namely, renal clear cell carcinoma, renal papillary adenocarcinoma, renal chromophobe cell carcinoma and collecting duct carcinoma, accounting for 60%~85%, 7%~14%, 4%~10% and < 1% of cases, respectively.^2^ The enhanced permeability and retention (EPR) effect enhances permeability and retention and is characterized by rich blood vessels, a wide gap between vascular walls and poor structural integrity of the solid tumor tissue, resulting in high selective permeability and retention of macromolecular substances and lipid particles.^3^ Long-term high blood pressure promotes the formation of atherosclerosis, which may affect the EPR effect. In this study, the enrichment of polymer nanomicelles (loaded with prolonium iodide) in renal tumor specimens was determined using CT iodine-based material decomposition imaging to explore whether blood pressure has an effect on the EPR effect in renal cell carcinoma.

## METHODS

A total of 80 patients with renal carcinoma treated at the Department of Urology at the Affiliated Hospital of Hebei University from October 2019 to October 2020 were analyzed retrospectively. The diagnosis of renal clear cell carcinoma was confirmed by preoperative auxiliary examinations, such as ultrasonography and CT combined with postoperative pathological analyses. All patients underwent laparoscopic radical nephrectomy for renal carcinoma, and anticoagulation treatment was performed on the resected kidney specimens. Micelles were perfused within the specified time (1 h, 2 h, 3 h, 4 h, 5 h and 6 h), and the enrichment of micelles in renal carcinoma foci was determined by CT scanning.

### Ethical approval:

This study was approved by the Institutional Ethics Committee of the Affiliated Hospital of Hebei University (No: HDFY-LL-2019-021) on June 9, 2019; and written informed consent was obtained from all participants.

### Inclusion criteria:

- Patients with renal tumor diagnosed based on symptoms, signs and imaging examinations;
- Patients with RCC (pathological stage, T1NOM0) confirmed by postoperative pathology;
- Patients with confirmed hypertension and a medical history.

### Exclusion criteria:

- Patients with other related complications, such as diabetic nephropathy, nephritis caused by various causes, nephrotic syndrome, metabolic syndrome, and uremia;
- Patients with renal masses originating from other causes, such as renal hemangiomas, renal hamartomas, and renal cysts;
- Patients with secondary hypertension, such as that resulting from adrenal gland diseases (e.g., pheochromocytoma);
- Patients with severe cardiopulmonary disease and those with intolerance to surgery.

The data on the clinical diagnosis, treatment and analysis of relevant laboratory specimens were collected, and corresponding processing was conducted. Additionally, all the clinical records of the corresponding patients were read, and patients were divided into the experimental group and the control group according to their blood pressure level

Human renal tumor tissue specimens resected by surgery (4-7 cm in size with the renal capsule and renal artery and vein as intact as possible) were immediately preserved in a box at 4°C. The specimens were collected from tumor foci by needle aspiration biopsy, fixed in 10% formalin, and sent to the Department of Pathology for optical pathological examination to determine the histological type of renal carcinoma tissue and diagnose the disease. For the remaining tumor tissue specimens, the pinhole was blocked using a medical gelatin sponge to ensure that the micelles did not flow out from the pinhole during perfusion. After inserting the arterial cannula into the renal artery, the artery was ligated and fixed. The renal vein was connected to the collection bag. Twenty percent heparin saline at 4°C was injected via the renal artery to prevent the blood from clotting and blocking the blood vessel. The color of the fluid from the renal vein was similar to that of the input fluid. After washing, HTK preservation solution at 4°C was infused and the sample was stored in a sterile bag at 4°C for preservation.

Renal tumor tissue specimens preserved at 4°C were placed in a constant-temperature and constant-humidity perfusion device, and HTK preservation solution at 37°C was cyclically perfused via the renal artery using a peristaltic pump. When the temperature of the fluid from the renal vein reached 37°C, the perfusion was maintained for a certain period of time to achieve a degree of vasodilation in the tumor tissues close to that which occurs in vivo. The perfusion pressure was set at 60 cmH_2_O. The urine collector was connected at the broken end of the ureter.

Renal tumor tissue specimens were cyclically perfused with polymer nanomicelles (loaded with prolonium iodide). After perfusion, CT GSI mode was used to scan during perfusion and irrigation with normal saline until the iodine content in the normal tissue was close to 0. The data were imported into the image postprocessing workstation, iodine-based images were selected, and the region of interest of the images was measured (images were taken at 1 h, 2 h, 3 h, 4 h, 5 h and 6 h during perfusion). The iodine content in renal carcinoma tissues was measured, and then the enrichment of iodine in the renal carcinoma foci was determined.

### Observed indicators:

The peak EPR effect and the time needed to reach the peak were determined and statistically compared between the experimental group and the control group.

### Statistical methods:

Data were processed using SPSS 19.0. The data measured were expressed as *χ̄*±*S* and analyzed by a t test. The enumeration data were expressed as the rate (%) and tested by the c^2^ test. P< 0.05 was considered statistically significant.

## RESULTS

**Fig.1:**
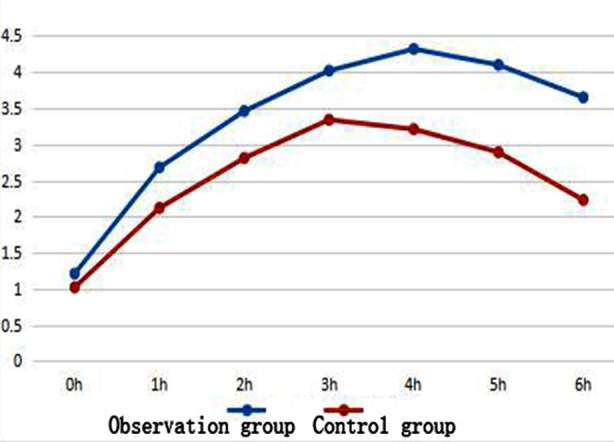
Change of peak with time (*χ̄*).

No significant differences were found in age, sex, location of kidney disease, tumor location or tumor size between the two groups (*p>* 0.05); thus, the groups were comparable (Table-I). EPR indexes of polymer nanomicelles (iodine enrichment): The peak (*χ̄*±*S*) of the EPR effect was 3.60±0.95 ug/cm^3^ in experimental group and 3.01±0.96 ug/cm^3^ in control group, respectively. There was significant difference between the two groups (*p<* 0.05). The time (h) to the peak of the EPR effect in experimental group was 3.76±0.75 hour and 3.82±0.93 h in control group, respectively. No statistically significant difference was found in the time to the peak of the EPR effect between the two groups (*p>* 0.05, Table-II). The change in the EPR peak (*χ̄*) over time is shown in Fig.1.

**Table-I:** Comparison of baseline data between the observation group and the control group (*χ̅*±*S*).

Clinical parameters	Observation group (n = 40)	Control group (n = 40)	p
Age (years)	54.89±7.22	55.18±7.66	>0.05
Maximum tumor diameter (cm)	3.46± 1.10	3.62± 0.82	>0.05
Systolic pressure	160.90±13.24	124.52±6.95	<0.05
Diastolic pressure	101.76±8.26	83.86±4.55	<0.05
***Gender [n (%)]***			
Male	28(70)	28(70)	>0.05
Female	12(30)	12(30)	>0.05
***Tumor location [n (%)]***			
Left	26(65)	24(60)	>0.05
Right	14(35)	16(40)	>0.05
Upper pole	20(50)	18(45)	>0.05
Medium pole	8(20)	10(25)	>0.05
Lower pole	12(30)	12(30)	>0.05

**Table-II:** Comparison of the EPR peak and time to the peak between the two groups (*χ̅*±*S*)

Group	n	Peak(ug/cm^3^)	Time to peak (h)
Experimental group	40	3.60±0.95	3.76±0.75
Control group	40	3.01±0.96	3.82±0.93
t		2.81	0.33
p		<0.05	>0.05

## DISCUSSION

The EPR effect of tumor is one of the most important theoretical bases for the targeted transport of Nano drugs.^3^ In recent years, the development of nanotechnology has changed the basis of the diagnosis, treatment and prevention of diseases. Recently developed polymer-based MA molecular drugs (polymer conjugates, micelles), liposome-encapsulated anticancer drugs and novel nanodevices such as carbon nanotubes and quantum dots are at the forefront of novel anticancer nanodrug treatments. The EPR effect links the progression of nanotechnology with the progression of the knowledge of tumor and vascular biology, marking the beginning of a new era.^4^ A large number of studies have shown that nano drugs have good enrichment and ideal therapeutic effect in experimental animal tumor models, but most of the research results have not been applied in clinical practice, and the therapeutic effect has not been obvious.^5^ The reason may be that there are great differences between experimental animal tumor models and human solid tumors. We constructed the experimental animal tumor model and the human isolated renal cell carcinoma specimen model, and used the in vitro perfusion method to simulate the blood circulation to study the relationship between the experimental animal tumor model and the EPR effect of human solid tumor. Studies have shown that human renal cell carcinoma also has EPR effect.^6^,^7^ The in vitro tumor perfusion model we constructed effectively avoids the influence of immune clearance and other factors in blood circulation, creates conditions for the study of human EPR effect, and also provides more reliable theoretical basis for the design and improvement of nano drugs.

Hypertension is a psychosomatic, chronic, and complex multifactorial disease that causes obvious changes in blood vessels. The approach in this study surpasses that of animal models by using polymer nanomicelles to investigate the EPR effect in human renal tissue specimens in vitro. In this experiment, a difference was found in the iodine enrichment measured in renal tumor specimens in vitro. The peak of the EPR effect was higher in the experimental group than in the control group, and the difference was statistically significant. Therefore, hypertension has a certain influence on iodine enhancement, namely, the EPR effect of polymer nanomicelles. The vascular endothelial barrier is indispensable for the regulation of cells, cytokine spillover into the blood and for the maintenance intravascular homeostasis. The loss of endothelial barrier integrity, endothelial cell proliferation disorder and enhanced inflammatory cell infiltration are common characteristics of the pathogenesis of vascular diseases.^8^ Vascular remodeling is the main determinant of changes in the lumen, including changes in cells of the vascular wall cell, the extracellular matrix structure and morphology.^9^ As a result, hyaline angiopathy and atherosclerosis occur, leading to a reduced inner diameter of blood vessels, thickened vascular wall and decreased vascular permeability. However, the metabolism of blood vessels is vigorous, and tumor cell proliferation is rapid. Therefore, the number of nanomicelles passing through the blood vessels in the tumor area increases over time; thus, the EPR effect is enhanced over time.^10^,^11^ The differences between tumor blood vessels and normal blood vessels are as follows:

1) The endothelial gap is very wide in tumor vessels; Upcin B et al. and Davis GE et al. found that the endothelial surface was porous by analyzing the gap between endothelial cells and found that they are surrounded by discontinuous cells.^12^,^13^
2) The blood vessel functions are lost in tumor cells and the lack of functional lymphatics in tumor tissue leads to the accumulation of more macromolecules or nanoparticles in the stroma of tumor tissue rather than in normal tissue.^14^

The solute level of tumor tissues is high. When interstitial colloid osmotic pressure increases, it promotes low-molecular-weight components and macromolecules to enter the tumor tissue from the tumor blood vessels. Although small molecules can freely enter and exit the blood vessels from normal tissues and tumor tissues, macromolecules cannot be effectively transferred to the lumen side of the capillaries, which results in the significant accumulation and retention of anticancer nanodrugs in tumor tissues. Maeda H found that if the systolic blood pressure was increased from 100mmHg to 150mmHg, the distribution of SMANCS in tumor tissue would be greatly increased. If the hypertension was maintained for about 15 minutes, the drug distribution in tumor tissue would be increased by 2-3 times^15^. We know that, in addition to VPF or VEGF, many other vascular mediators can further enhance the EPR effect, including ATII, BK, NO, peroxynitrite (ONOO2), matrix metalloproteinases (MMPs, or collagenase) and PGs.^16^,^17^ The smooth muscle layer of tumor blood vessels is absent, which makes them unable to sense neurotransmitters and contract. Therefore, vasoconstrictors (such as AT-II) can inhibit the effect of high blood pressure on tumor blood vessels. AT-II can promote drug delivery to other tumor tissues, and the hemodynamics of tumor tissues are different from those of normal tissues or organs.^16^ Moreover, there is no dynamic balance of blood flow in tumor tissues. Following infusion with AT-II, tumor blood flow and blood pressure gradually increases after the administration of tumor-targeted drugs, which shows that AT-II-induces vascular permeability, increases the diameter of tumor blood vessels and widens the endothelial cell gap, thus promoting more drug leakage. In normal blood vessels, AT-II limits the connection between endothelial cells and other cells, decreases drug leakage and reduces side effects,^17^,^18^ such as myelosuppressive and gastrointestinal side effects.

The EPR effect allows for the delivery of selective anticancer nanodrugs due to the anatomical and pathophysiological defects of tumor blood vessels.^19^ The use of biocompatible nanodrugs and existing nanotechnology to develop EPR effects and deliver cytotoxic anticancer drugs is a more effective and safer cancer treatment method. It is necessary to understand and control the factors affecting the EPR effect, which improves the selective targeting of anticancer nanodrugs to tumor tissues.^20^ The EPR effect can be enhanced in many ways, such as the use of vascular mediators, which can cause transient changes in the diameter, wall structure and function of the blood vessels adjacent to the tumor area.^21^-^23^

### Limitations of this study:

The length of history of patients with hypertension may have an impact on the results. In the next step, multivariate analysis can be carried out in combination with patient history, the presence or absence of metabolic syndrome and other factors.

## CONCLUSION

The iodine enrichment amount measured in the isolated renal tumor samples was higher in the hypertensive group than in the non hypertensive group, so it can be considered that hypertension has a certain impact on the iodine enrichment amount, that is, EPR effect of polymer nano micelles.

### Authors’ Contributions:

**ZC** and **WS** designed and conceived the research.

**TM and WY** Literature search and analyzed the data.

**WS** is responsible and accountable for the accuracy and integrity of the work. All authors reviewed the manuscript and approved the final manuscript.
